# The Potential Role of CD44 and CD133 in Colorectal Stem Cell Cancer

**DOI:** 10.7759/cureus.30509

**Published:** 2022-10-20

**Authors:** Wael Abdou Hassan, Mohamad Ayham Muqresh, Mohamed Omer

**Affiliations:** 1 Department of Basic Sciences, College of Medicine, Sulaiman Al Rajhi University, Al Bukayriyah, SAU; 2 College of Medicine, Sulaiman Al Rajhi University, Al Bukayriyah, SAU

**Keywords:** prognosis, therapy, diagnosis, biomarker, cd133, cd44, cancer stem cells, colorectal cancer

## Abstract

Colorectal cancer (CRC) is the most preventable malignancy globally, with a high mortality rate. Cancer stem cells (CSCs) are found previously in multiple types of cancer; CRC is one of them, and it has been correlated with several biomarkers. The two most essential markers related to colorectal CSCs are CD44 and CD133, which play a significant role in diagnosis, treatment, and prognosis. Unfortunately, the CSCs with positive CD44 and CD133 biomarkers illustrated an alarming prognosis. Several trials were trying to target those markers to improve the prognosis and cure. We aimed to review the papers that relate to the two markers in terms of diagnosis, treatment, and prognosis.

## Introduction and background

Introduction

Colorectal cancer (CRC) is a well-known preventable cancer among other different cancer cells. It is the third most common cancer that causes death worldwide [1]. These days, CRC is considered the third most common cancer diagnosis in the USA due to increasing awareness of early screening for colorectal cancer and the widespread availability of colonoscopy in medical centers [2]. The early diagnosis of CRC has been associated with improving prognosis [3]. Unfortunately, CRC showed increasing incidence and mortality among young individuals below 50 years old [4]. Colorectal cancer is caused by genetic mutations, including those in the adenomatous polyposis coli (APC), deleted in colon cancer (DCC), K-Ras, p53, B-Raf proto-oncogene serine/threonine kinase (BRAF), and microsatellite instability [4]. The recent management plan depends completely on the stage of cancer and whether the cancer cells have metastasized to a different area or not; the approaches include surgery, endoscopic ablation, and adjunctive treatment such as radiotherapy and chemotherapy [5].

Cancer stem cell (CSC) theory is not a modern model. The model could be traced to the 19th century when leukemia was the first malignancy of CSCs discovered, but it was not until 1970 that cancer stem cells were heavily investigated [6,7]. The majority of tumors contain CSCs, which are self-renewable cell types linked to tumor development, growth, resistance to treatment, recurrence, and metastasis following treatment [8]. Cell surface marker expression is dependent on the tumor type, which is used to identify CSCs [5,9]. Cancer-associated fibroblasts are one of the most important cells for promoting the differentiation of CSCs and the dedifferentiation of non-CSCs toward developing a CSC-like phenotype. Necessary signals for preserving self-renewal in CSCs include WNT/β-catenin, transforming growth factor-β (TGF-β), Hedgehog, and Notch [10].

One of the well-known characteristics of CSCs is the ability to protect themselves from traditional chemotherapeutic drugs, imparting a poor prognosis and decreasing the overall survival among the patients [7]. The primary and secondary resistance of cancer cells is one of the critical reasons that limit chemotherapy's efficacy; primary resistance means the tumor is not responding to the therapy, while secondary resistance means the tumor responded well to the treatment and then developed resistance [11]. The studies showed several mechanisms of how CSCs are not responding to the drug, raising the call for researchers to conduct studies about reducing the resistance [12]. This study aims to review the articles that include the role of CD44, and CD133 in colorectal CSCs in terms of diagnosis, therapy, and diagnosis.

Material and methodology

In this review paper, we searched PubMed, Google Scholar, and the Cochrane Database of Systemic Reviews from 2010 until 2022. During searching through different databases, we utilized multiple combinations of MeSH words “CD44,” “CD133,” “Colorectal cancer,” “Cancer stem cells,” “Treatment,” “prognosis,” and “Diagnosis”. We found 25 articles as a total number of studies. Authors combine the article’s findings in one shared sheet on Google Drive and assess their qualifications according to our inclusion criteria, and eliminate papers that met our exclusion criteria, which end up with 12 papers as a total number.

Our inclusion criteria were interventional studies, review studies, and original’s studies that have been published between 2010 and 2022. We exclude non-English papers, multiple limitations papers, and non-open access with difficulty reaching out to the corresponding author. We have evaluated and summarized the discussion part of each article and the conclusion.

## Review

The well-known biomarkers of cancer stem cells in different organs

Several studies have provided the most prominent biomarkers that could play an essential role in the diagnostic, therapeutic, and prognostic approach to CSCs. More importantly, some biomarkers have been linked to a specific tumour, for example, CD44, which is found mainly in colorectal and haematological cancer [13]. In the section below, we focus on the markers strongly associated with CSCs.

CD44 and CD133

CD44 is a non-kinase antigen found expressed on embryonic stem cells in various cell types. Recently, CD44 has been used to recognize the CSCs for different kinds of cells, such as lung cancer, breast cancer, colon cancer, haematological cancer, and other cancers still under investigation [14]. CD44 is linked to a significant potential biomarker for diagnosing colon cancer stem cells, which arise an alert for developing a therapy that targets CD44 to reduce mortality among colorectal cancer patients [15]. CD133 is a five-transmembrane glycoprotein, first discovered in 1997 and found to be expressed on hematopoietic stem and progenitor cells generated from blood, fetal liver, and bone marrow [16]. Various studies showed that CD133 is a potential marker for CSCs and is found in several tumours, including colorectal, brain, kidney, lung, pancreas, bone, and ovary [13].

CD123 and CD33

CD123 and CD33 are the classical markers in haematological malignancy. CD123 is expressed in leukaemia CSCs and correlates with increased proliferation and differentiation. CD33 was found to be expressed in acute myeloid leukaemia (AML) and can be targeted by the gemtuzumab ozogamicin agent [17].

EpCAM (CD326) and Oct-3/4

EpCAM is a marker discovered in colon cancer and consists of glycoprotein. EpCAM is expressed in different types of epithelial tissues [18,19], as well as in CSCs such as colorectal and hepatocellular carcinoma [20,21]. AlShamaileh et al. (2017) conducted a study preclinically targeting EpCAM in colorectal cancer [22]. Oct-3/4 is found to be expressed in embryonic stem cells and maintains the self-renewal of embryonic stem cells [23]. In addition to its role as a prognostic marker in colorectal cancer [24], some studies found that Oct-3/4 is involved in other tumours, such as breast cancer metastasis, osteosarcoma, and cervical cancer [25-27].

The potential role of CD44 in colorectal cancer stem cells

CD44 as a Surface Cell Marker

CD44 plays a vital role in cell-cell reaction, migration, and cell adhesion [14,28]. It was found that CD44 expression worsens the prognosis due to increased tumour aggressiveness and high resistance to chemotherapy and radiotherapy [29]. Targeting CD44 will reduce cell proliferation, migration, and invasion and promote apoptosis; hence, overall survival will increase [30].

CD44 as a Therapeutic Target in Colorectal Cancer

The management of CRC requires multiple disciplinary team members. Surgery with adjuvant chemotherapy is usually utilized to manage CRC, especially in high-risk patients or those with advanced stages [31]. However, such treatment options still do not provide the best results due to the primary resistance, recurrence, toxicity of the treatment, its effect on life expectancy, and not targeting cancer stem cells [32]. Several studies believed that CSCs demonstrated the responsibility for drug resistance, relapse, and recurrence [12,32,33].

Accumulating papers illustrated great efforts to attack and target the well-known markers of colorectal cancer. Lee et al. (2017) successfully built and constructed a CD44-short hairpin RNA (shRNA) recombinant adenoviral model in a way that could knock down CD44 by adenoviruses [30]. Virotherapy is a known method that is still under the study process, and it can be used to target cancer cells without affecting healthy cells [34]. Lee et al. (2017) illustrated a surprising result using such shRNA, demonstrating a reduction in colorectal cancer cell proliferation, migration, and invasion by targeting several signalling pathways that control the tumour characteristics. For example, CD44-shRNA adenovirus blocked Akt phosphorylation, which is responsible for the survival of cancer cells, and inhibited GSK-3b, which regulates several proteins responsible for cell proliferation. It also targeted Wnt singling that downregulates β-catenin, which is responsible for cell adhesion and tumour invasion. In addition, there was an expression reduction of Bcl-2 and Bcl-xL. In conclusion, CD44-shRNA adenovirus might affect colorectal cancer cells and induce apoptosis among cancerous cells by attacking CD44 [30].

Tsunekuni et al. (2019) showed that trifluridine (FTD) is effective in targeting CSCs expressing proteins CD44 and CD133 [35]. Limagne et al. (2019) showed that FTD combined with tipiracil (TPI) increase the bioavailability of FTD [36]. FTD works by inhibiting thymidylate synthase (TS), which is the enzyme that elaborates the conversion of deoxyuridine monophosphate (dUMP) to deoxythymidine monophosphate (dTMP); so inhibiting TS may lead to DNA damage of positive CD44 and CD133 cells. FTD is used in clinical practice in case of refractory or metastatic colon cancer [35].

Fu et al. (2020) stated that atovaquone, which usually treats malaria infection, can be used against positive CD44 and EpCAM cancer cells. Atovaquone stopped the proliferation by blocking S-phase under hypoxia, which causes apoptosis to CSCs, but the authors stated that the study has some limitations during the trial process, which may affect the apoptotic rate; this calls for further research to be conducted [37].

CD44 as a Prognostic Marker in Colorectal Cancer

Xia et al. (2016) and Spelt et al. (2018) illustrated that CD44 is one of the promising markers that will effectively assess the prognosis for colorectal CSCs [38,39]. Ozawa et al. (2014) found positive CD44 alone showed poor survival overall. And the combination of positive CD44 and CD133 showed the same overall survival result as positive CD44 alone, in which CD44 is effective in assessing the prognosis of colorectal cancer [40].

The potential role of CD133 in colorectal cancer stem cells

CD133 as a Diagnostic Marker

Lim et al. (2014) demonstrated that CD133 could be a valid diagnostic marker, as they found that more than 3% of positive CD133 was highly associated with the sphere formation of colorectal cancer cells. Furthermore, it illustrated that more than 3% of positive CD133 had a radically decreased overall survival rate [41].

CD133 as a Therapeutic Target in Colorectal Cancer

Acikgoz et al. (2020) demonstrated that triptolide (TPL) treatment could be effective against positive CD133 as well as CD44 colon cancer stem cells. Triptolide is utilized in several tumours such as of the prostate, breast, colon, and lung. TPL is a cytotoxic drug that showed an effect on positive CD133 and CD44 cancer cells by inhibiting the proliferation of colon cancer which leads to cell cycle arrest and induces apoptosis. However, it inhibited the spheroid formation, migration, and invasion of cancer stem cells. TPL causes apoptosis because it activates caspases, which regulate the apoptotic process [42].

Ning et al. (2016) tried to target CD133 with antibody-conjugated SN-38-loaded nanoparticles, which illustrated a significant efficacy in stopping the growth and reducing the recurrence in positive CD133 colorectal cancer stem cells [43]. Zhao et al. (2015) developed an antibody called MS133 that showed a good result by having an affinity and cytotoxicity against CD133. The trial of MS133 was done on mice and illustrated the eradication of cancer stem cells from them. Furthermore, it showed that CD133 overexpression in colorectal cancer stem cells was significantly suppressed by the MS133 antibody [44].

## Conclusions

In conclusion, CD44 and CD133 are found to be well-known biomarkers in colorectal CSCs. They showed their importance in predicting the prognosis of colorectal cancer. The paper showed promising outcomes by targeting these two markers, which may enhance the overall survival rate and prognosis of the patient in the future. However, most of these studies were conducted preclinically. Hence, we call for further future research and investigations to be conducted for the purpose of identifying the most effective treatments that specifically target CD44 and CD133 in colorectal cancer.
